# Whole-lung Low Dose Irradiation for SARS-Cov2 Induced Pneumonia in the Geriatric Population: An Old Effective Treatment for a New Disease? Recommendation of the International Geriatric Radiotherapy Group

**DOI:** 10.14336/AD.2020.0506

**Published:** 2020-05-06

**Authors:** Pedro C Lara, Nam P Nguyen, David Macias-Verde, Javier Burgos-Burgos, Meritxell Arenas, Alice Zamagni, Vincent Vinh-Hung, Brigitta G Baumert, Micaela Motta, Arthur Sun Myint, Marta Bonet, Tiberiu Popescu, Te Vuong, Gokula Kumar Appalanaido, Lurdes Trigo, Ulf Karlsson, Juliette Thariat

**Affiliations:** ^1^Department of Radiation Oncology, Hospital Universitario San Roque, Fernando Pessoa Canarias Las Palmas University, Las Palmas, Spain.; ^2^Department of Radiation Oncology, Howard University, Washington D.C., USA.; ^3^Department of Radiation Oncology, Sant Joan de Reus University, University Rovira I Virgili, Tarragona, Spain.; ^4^Radiation Oncology Center, Department of Experimental, Diagnostic and Specialty Medicine, Sant’Orsola-Malpighi Hospital, University of Bologna, Bologna, Italy.; ^5^Department of Radiation Oncology, University Hospital of Martinique, Martinique, France.; ^6^Institute of Radiation Oncology, Cantonal Hospital Graubuenden, Chur, Switzerland.; ^7^Department of Radiation Oncology, ASST Papa Giovanni XXIII, Bergamo, Italy.; ^8^Department of Radiation Oncology, Clatterbridge Cancer Center, Liverpool, United Kingdom.; ^9^Department of Radiation Oncology, Arnau de Vilanova University Hospital, Lleida, Spain.; ^10^Department of Radiation Oncology, Prof. Dr. Ion Chricuta Oncology Institute, Cluj-Napoca, Romania.; ^11^Department Of Radiation Oncology, McGill University, Montreal, Canada.; ^12^Department of Radiation Oncology, Penang Adventist Hospital, Penang, Malaysia.; ^13^Department of Radiation Oncology, Instituto Portuges de Oncologia Porto Francisco Gentil E.P.E, Porto, Portugal.; ^14^Department of Radiation Oncology, International Geriatric Group, Washington D.C., USA.; ^15^Department of Radiation Oncology, Baclesse Cancer Center, Caen, France.

**Keywords:** elderly, SARS-Cov 2, LDRT, treatment, inflammation

## Abstract

A cytokine storm induced by SARS-Cov2 may produce pneumonitis which may be fatal for older patients with underlying lung disease. Hyper-elevation of Interleukin1 (IL-1), Tumor necrosis factor-1alfa (TNF-1 alfa), and Interleukin 6 (IL-6) produced by inflammatory macrophage M1 may damage the lung alveoli leading to severe pneumonitis, decreased oxygenation, and potential death despite artificial ventilation. Older patients may not be suitable candidates for pharmaceutical intervention targeting IL-1/6 blockade or artificial ventilation. Low dose total lung (LDTL) irradiation at a single dose of 50 cGy may stop this cytokine cascade, thus preventing, and/or reversing normal organs damage. This therapy has been proven in the past to be effective against pneumonitis of diverse etiology and could be used to prevent death of older infected patients. Thus, LDRT radiotherapy may be a cost-effective treatment for this frail patient population whom radiation -induced malignancy is not a concern because of their advanced age. This hypothesis should be tested in future prospective trials.

The corona virus disease 19 (COVID 19) induced by SARS-CoV-2 is a pandemic affecting all countries in the world. Although most infected individuals experienced mild or no respiratory symptoms, in older patients (65-year-old) or older with underlying co-morbidity, COVID 19 may induce severe lung inflammation leading to acute respiratory distress syndrome (ARDS), respiratory failure, and death despite artificial ventilation. To date, by April 16^th^, 2020, 137,078 deaths have been reported mostly affecting older patients. Infected patients who are 80-year-old or older comprised half of the death rate. Pathology report of asymptomatic infected patients who inadvertently underwent lobectomy for early stage lung cancer revealed an intense inflammatory reaction characterized by mononuclear cells infiltration, fibrin exudates, multinucleated giant cells, and thickened alveoli secondary to proliferating interstitial fibroblasts and type II pneumocyte hyperplasia [1]. Other report from patients with more advanced pulmonary disease also corroborated this finding in addition to elevation of inflammatory markers such as ferritin and Dimer D [2]. Thus, an effective strategy to improve mortality rate of older patients should target the inflammatory cycle either with medications or with low dose radiotherapy to the whole lung. As an international organization devoted to older cancer patients, the International Geriatric Radiotherapy Group (IGRG) (www.igrg.org) would like to contribute to the global effort against COVID [3]. This review addresses the potential of radiotherapy as a cost-effective treatment for older patients with multiple co-morbidities which preclude them from intensive care unit (ICU) admission and artificial ventilation.

## Pathophysiology of COVID 19 pneumonitis

It is postulated that the intense inflammation observed in the lung parenchyma (ARDS) is due to an inflammatory cascade characterized by elevation of Interleukin1 (IL-1), tumor necrosis factor 1 (TNF-1alfa) and interleukin 6 (IL-6) produced by inflammatory macrophage M1 [4]. This inflammatory profile was also reported in previous pandemics such as SARS-Cov and MERS-Cov [5].

Acute respiratory distress syndrome is also named as the macrophage activation syndrome (MAS) because of the crucial role of macrophages in the generation of cytokines storm responsible for irreversible lung injury.

Macrophages have been classified into classically activated (M1) and alternatively activated (M2) macrophages, based mainly in the differential protein secretion, and roles in host defense. Beyond these simple stratifications, a continuum of macrophage polarization likely exists. The M1 classically activated macrophage phenotype, is induced by proinflammatory cytokines, such as Interferon (INT-γ) and Lypopolysacchrides (LPS), and its activation leads to the production of proinflammatory IL-1β, IL-12, TNF-α, and inducible nitric oxide synthase (iNOS). The M2 alternatively activated macrophage phenotype, is induced by the Th2 cytokines, IL-4 and IL-13, and produces IL-10, a well-known anti-inflammatory cytokine. Macrophages can modify their functional phenotype (macrophage polarization) due to micro-environment cytokines and other factors [4].

Under normal circumstance, macrophages play a major role in the defense against infectious agents such as bacteria or virus. The normal lung parenchyma contains alveolar macrophages which are located in the air-tissue interface [6]. The predominant macrophages are of M2 subtype which are responsible for normal immunosuppression [7]. However, in response to a foreign antigen such as SARS-CoV2, peripheral blood monocytes are recruited into the alveoli where they differentiate into M1 macrophage which produce inflammatory cytokines such as IL-1, IL-6, and IL-18 which in turn attract neutrophils cells into the alveoli to fight the infection, leading to infection clearance through reactive oxygen species (ROS) and phagocytosis. Once the pathogens are eliminated, M1 macrophages are transformed into the M2 subtype which reduce the inflammatory lung reaction until the lung parenchyma reverts to its normal state [8]. However, in abnormal circumstances such as ARDS or autoimmune diseases, the hyper inflammatory state continues unabated leading not only to lung damage but to multiple normal organs destructions such as kidney failure, cardiac damage and ultimately death [9]. Persistence of inflammatory neutrophils in the alveoli along with increased concentration of ROS and TNF are thought to contribute to lung injury [10].

The role of macrophages in inducing cytokines storm is well illustrated in past coronavirus epidemic [5]. Other studies also corroborated their role in the generation of permanent lung injury [11]. Their function was also highlighted in auto immune disease such as rheumatoid arthritis (RA) [12]. The inflammatory role of imbalance macrophage function has been illustrated in the clinical presentation of COVID-19 ARDS characterized by increased ferritin, C-reactive protein (CRP], D dimers levels and inflammatory cytokines [5,13]. Chen *et al* [13] reported increased elevation of those biomarkers among patients with severe COVID-19 clinical manifestations. In addition, the multiple organs failure observed among infected patients who died after admission to the ICU is in favor of the hypothesis of cytokines storm as the culprit of COVID-19 ARDS [14].

Perhaps, the most compelling clinical evidence for the role of cytokines storm in the pathogenesis of ARDS came from clinical data on plasmapharesis or therapeutic plasma exchange (TPE) [15]. Among 20 patients with hypotension and sepsis requiring high dose vasopressors, TPE reversed the clinical course which is frequently fatal. There was a significant reduction of the inflammatory cytokines following TPE which paralleled patient recovery. In addition TPE was also effective in the treatment of interstitial pneumonitis in patients suffering the complications of lupus erythematosus (LE), an auto-immune disease [16-18] Taking together, any effective therapeutic intervention needs to break this vicious cycle of cytokines storm either through their modulating effect on macrophages function, and/or inflammatory cytokines [19]

### Pharmacology intervention to reduce COVID-19 induced cytokines storm

Although controversial, many clinical trials are currently conducted to assess the efficacy of many drugs for the treatment of COVID-19 induced ARDS. For example, high dose steroids have been advocated to suppress the inflammatory state even though its efficacy remains dubious [20]. Other medications that may impair macrophages function such as the antimalarial drug hydrochloroquine [20], or may act like interleukin antagonists such Anakinra (interleukin-1 receptor antagonist), and Tocilizumab (interleukin-6 receptor antagonist), [16,21], or downstream signaling inhibitors such Baricitinib (JAK1/JAK2 inhibitor) [22] are currently under investigation for treatment COVID-19 ARDS. However, until the results are published, their efficacy remains to be seen.

Low dose radiotherapy (LDRT) is an effective therapy for inflammatory diseases including pneumonia. Long before high dose radiotherapy became a standard treatment for cancer, LDRT has been employed to treat benign diseases because of its anti-inflammatory effect. Indeed, LDRT was a popular and effective treatment for a myriad of inflammatory or infectious disease ranging from arthritis to sinusitis, ear infection, and potentially deadly infection such as pneumonia and gas gangrene [23-30]. A single treatment of 20 cGy to 200 cGy has been reported to be effective in inducing fast and lasting effects for those conditions [29]. Single doses over 200 cGy, are now abandon in the treatment of this situations, as it has been shown that would increase lung pneumonitis by activation of the cytokine release including TGFβ, leading to interstitial collagen deposition [31] . As an illustration, up to 90% of patients suffered from shoulder bursitis had immediate relief of pain and stiffness following LDRT [26]. Powell et al [32] reported immediate relief of dyspnea and fever following a single fraction of LDRT for patients with lobar pneumonia in 1933. Only 5 out of those 104 patients died after lung LDRT which was remarkable as Penicillin was just discovered by Sir Alexander Fleming in 1928, and only became available for treatment in 1944. Other authors also corroborated the effectiveness of LDRT for pneumonia, bronchopneumonia and interstitial pneumonia in the same era [33-35]. In animal experiment, LDRT also corroborated the beneficial effect of LDRT in virus induced pneumonia [36]. However, LDRT went out of vogue when antibiotics and anti-inflammatory medications became effective treatment and out of concern for long-term malignancy induced by LDRT [37]. Thus, success of LDRT for those diseases should be based on its anti-inflammatory modulation at the molecular level.

### Cellular physiology of LDRT

A single dose of 50 cGy has been reported to reduce synovial inflammation in mice suffering from arthritis [38]. Compared to sham irradiation, LDRT at a single dose of 30 cGy significantly reduced blood leucocytes counts and leucocytes adhesion in mice with lipopolysaccharide (LPS) induced peritonitis [39]. A similar experience with a single 50 cGy fraction produced significant reduction of IL-1β, a pro-inflammatory cytokine produced by activated macrophages [40]. In another experiment with the same radiation dose, there was a significant function reduction of activated of M1 macrophage suggesting the central role of macrophage in LDRT cytokines modulation [41]. Indeed, production of TNF-1α, IL-1β, and IL-6 produced by M1 macrophage was significantly reduced following LDRT [42]. As a result, significant reduction of ROS was observed leading to less destruction of normal tissues [43]. In addition, there were a shift of macrophages from the M1 to the M2 subtype after LDRT which may further decreased the inflammatory process [44]. A marked increase of M2 macrophages was observed following a single dose of 200 cGy suggesting that those later cells are more resistant to LDRT compared to the M1 subtype [45]. Other studies also corroborated the effect of LDRT on the suppression of inflammatory cytokine IL-6 in arthritic mice [46].

Taken together, those studies suggested that LDRT decreased the inflammatory process through modulation of M1 and M2 macrophages to decrease the cytokines storm and could be beneficial to COVID-19 induced pneumonitis.

Damage induced by low-dose radiation on progenitors of inflammatory cells, and increased cancer risk, is still a major concern today. This concern comes from predictions of the linear non-threshold (LNT) model for accidental radiation exposures. Epidemiological studies in elderly patients treated, showed no increased cancer-risk at low-dose radiotherapy [37]. Furthermore, at the present scenario we are facing either the real risk of dying from the SARS-CoV-2 IL-6 pneumonia and the advanced age of the patients at such risk, would make irrelevant such concerns.

### Proposed protocol for LDRT as a potential treatment for COVID-19 induced pneumonitis

The COVID-19 pandemic forced physicians to a painful choice of patient selection for available treatment because of limited resources in many countries [47]. Older patients (>65 years old) with multiple co-morbidity may not be candidates for hospital intensive care unit admission when they developed pneumonitis [48]. Thus, they are faced with an almost certain death when provided with home supportive care. As LDRT has been effective in the past to treat pneumonia, a single dose of 50 cGy to the whole lung is advocated for those patients. This treatment is well tolerated and could be given as an outpatient provided that appropriate measures are taken to protect health care workers and other patients. The risk of long-term radiation-induced malignancy is mitigated because of those patients’ short life expectancy. To our knowledge, current clinical trials are undergoing to test the efficacy of LDRT for COVID-19 pneumonitis. However, with our large network of over 800 radiation oncology institutions in 126 countries, LDRT would provide significant access to treatment for many countries with scarce resources and to acquire data for further management of the geriatric population affected by this pandemic.

### Conclusion

Older patients who developed COVID-induced pneumonitis may benefit from LDRT to the whole lung, to improve their quality of life and survival. This cost-effective treatment should be tested as a clinical trial for all countries to decrease the burden of the hospital system which is currently being overwhelmed by the increased number of infected patients.
